# Opening the conformation is a master switch for the dual localization and phosphatase activity of PTEN

**DOI:** 10.1038/srep12600

**Published:** 2015-07-28

**Authors:** Hoai-Nghia Nguyen, Jr-Ming Yang, Takafumi Miyamoto, Kie Itoh, Elmer Rho, Qiang Zhang, Takanari Inoue, Peter N. Devreotes, Hiromi Sesaki, Miho Iijima

**Affiliations:** 1 Department of Cell Biology, The Johns Hopkins University School of Medicine, Baltimore, MD.

## Abstract

Tumor suppressor PTEN mainly functions at two subcellular locations, the plasma membrane and the nucleus. At the plasma membrane, PTEN dephosphorylates the tumorigenic second messenger PIP3, which drives cell proliferation and migration. In the nucleus, PTEN controls DNA repair and genome stability independently of PIP3. Whereas the concept that a conformational change regulates protein function through post-translational modifications has been well established in biology, it is unknown whether a conformational change simultaneously controls dual subcellular localizations of proteins. Here, we discovered that opening the conformation of PTEN is the crucial upstream event that determines its key dual localizations of this crucial tumor suppressor. We identify a critical conformational switch that regulates PTEN’s localization. Most PTEN molecules are held in the cytosol in a closed conformation by intramolecular interactions between the C-terminal tail and core region. Dephosphorylation of the tail opens the conformation and exposes the membrane-binding regulatory interface in the core region, recruiting PTEN to the membrane. Moreover, a lysine at residue 13 is also exposed and when ubiquitinated, transports PTEN to the nucleus. Thus, opening the conformation of PTEN is a key mechanism that enhances its dual localization and enzymatic activity, providing a potential therapeutic strategy in cancer treatments.

Phosphatidylinositol (3,4,5)-trisphosphate (PIP3) is a potent second messenger that drives many biological processes such as cell proliferation, growth, survival and migration^1^,^2^,^3^. In response to activation of a variety of receptors such as receptor tyrosine kinases and G-protein-coupled receptors by extracellular stimulation, PI3-kinases phosphorylate phosphatidylinositol (4,5)-bisphosphate (PIP2) to produce PIP3 at the plasma membrane. Phosphatase and tensin homolog (PTEN) dephosphorylates PIP3 to regulate PIP3 signaling^4^,^5^. In many cancers, PIP3 signaling is enhanced due to mutations in components of the signaling pathway, including EGF receptors, PI3-kinases, PTEN, Ras GTPases, and a major effector of PIP3, AKT^6^,^7^,^8^,^9^,^10^,^11^,^12^,^13^.

In addition to the plasma membrane, PTEN also localizes to the nucleus. Nuclear PTEN regulates DNA repair, genome stability and cell cycle progression^14^,^15^,^16^,^17^,^18^. Some studies have shown that these nuclear functions of PTEN do not involve PIP3 signaling and are independent of PTEN phosphatase activity^14^,^15^. The absence of nuclear PTEN is associated with aggressive cancers and Cowden syndrome^18^,^19^. Given that PTEN is a haploid-insufficient tumor suppressor, altering PTEN expression, localization and activity in cancer cells can be used to modulate its role. Especially, since PTEN is mainly located in the cytosol^13^,^20^,^21^,^22^,^23^, recruiting more PTEN to the plasma membrane and/or nucleus to stimulate its tumor suppressor functions may be an effective strategy to treat cancers.

PTEN interacts with lipids through its N-terminal PIP2-binding domain, its catalytic domain and its C2 domain^10^,^20^,^23^,^24^,^25^,^26^,^27^. Positively charged residues in the PIP2-binding and C2 domains have been proposed to recruit PTEN to the plasma membrane through associations with negatively charged membrane lipid head groups^21^,^22^,^28^,^29^. Using the heterologous expression system in which human PTEN is expressed and analyzed in the social amoebae *Dictyostelium*^22^,^23^,^30^, we previously identified a membrane-binding regulatory interface located on the surface of the human PTEN molecule, spanning the phosphatase and C2 domains^23^. This interface is masked by the inhibitory C-terminal tail, keeping most PTEN molecules in a closed conformation^21^,^23^,^31^. This intramolecular inhibition is controlled by phosphorylation of four serine/threonine residues (Ser_380_, Thr_382_, Thr_383_, Ser_385_) in the tail domain. A mutant PTEN (PTEN_A4_), in which these phosphorylation sites are substituted with alanines, is maintained in an open conformation and targeted to both the plasma membrane and the nucleus^21^. Furthermore, by engineering the membrane-binding regulatory interface, we previously generated an enhanced PTEN (ePTEN) which almost exclusively localizes to the plasma membrane and effectively suppresses PIP3 signaling^22^,^30^. Therefore, switching between the open and closed conformations provides not only a critical regulatory mechanism for the recruitment of PTEN to the plasma membrane, but also for its enzymatic activity. In contrast to the membrane recruitment, it is largely unknown how PTEN is transported to the nucleus.

## Results

### PTEN_A4_ and ePTEN redistribute to the nucleus when dissociated from the plasma membrane

**Figure 1:**
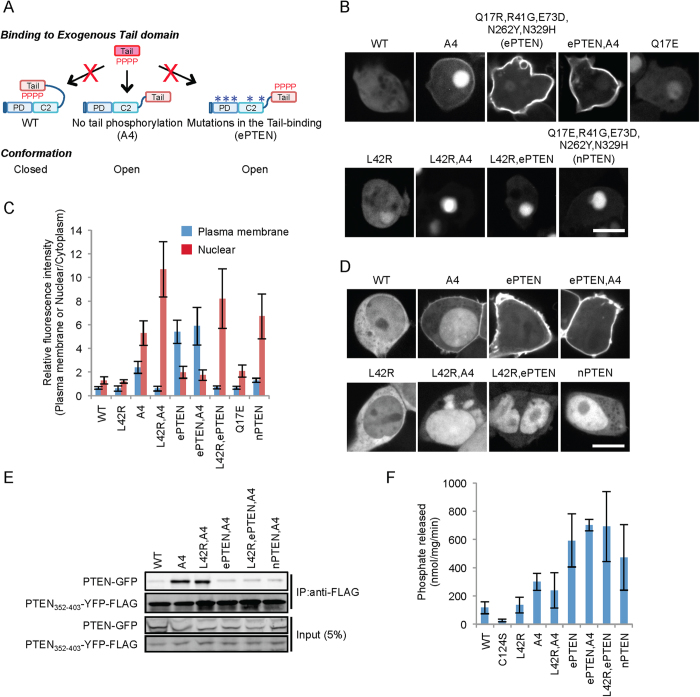
L42R redistributes PTEN_A4_ and ePTEN to the nucleus from the plasma membrane. (**A**) Associations between the core region (e.g. the phosphatase and C2 domains) and the tail of the same PTEN molecule (closed conformation) are maintained through the phosphorylation of the tail (indicated by “P”). In contrast, the core region of PTEN_A4_ dissociates from the tail (open conformation) and binds to an exogenously added tail domain^22^. ePTEN exists in an open conformation due to mutations in the core region (asterisks). Therefore, ePTEN cannot bind to the exogenously added tail^22^. (**B**) *Dictyostelium* cells expressing the indicated forms of PTEN-GFP were observed by fluorescence microscopy. Bar, 10 μm. (**C**) Intensity of GFP at the plasma membrane and in the nucleus was quantified relative to that in the cytosol. Values represent the mean ± SD (n ≥ 8). (**D**) HEK293 cells expressing the indicated forms of PTEN-GFP were observed by fluorescence microscopy. Bar, 10 μm. (**E**) Whole-cell lysates from *Dictyostelium* expressing the indicated PTEN-GFP proteins were incubated with PTEN_352–403_-YFP-FLAG expressed in HEK293 cells. PTEN_352–403_-YFP-FLAG was immunoprecipitated with beads coupled to anti-FLAG antibodies. (**F**) The indicated PTEN-GFP proteins were immunopurified from *Dictyostelium* cells (Fig. S2), and phosphatase activities were measured. Values represent the mean ± SD (n = 4).

To define mechanisms that localize PTEN to the nucleus and plasma membrane, we tested whether dissociation of PTEN from the plasma membrane drives translocation of PTEN into the nucleus. We used two mutant forms of PTEN, PTEN_A4_ and ePTEN, both of which are trapped in the open conformation^22^,^23^. While PTEN_A4_ carries mutations in the phosphorylation sites in the C-terminal tail, ePTEN has mutations in the core region (Fig. 1A)^22^,^23^. We expressed these PTEN variants as GFP fusions and examined their localization in *Dictyostelium* cells (Fig. 1B,C). PTEN_A4_ localized to the plasma membrane and nucleus while ePTEN predominantly associated with the plasma membrane due to a higher affinity for the membrane^22^. To dissociate PTEN_A4_ and ePTEN from the plasma membrane, we introduced a cancer-associated mutation, L42R (lysine changed to arginine at residue 42), which inhibits PTEN-membrane association^13^. While L42R alone did not alter the cytoplasmic localization of wild-type PTEN, introduction of L42R dramatically redistributed PTEN_A4_ and ePTEN to the nucleus with 2-4 fold increase in nuclear PTEN. The extent of nuclear PTEN_L42R,A4_ and ePTEN_L42R_ accumulation was similar to that of nPTEN^22^, which carries a single substitution near the PIP2 binding motif of ePTEN (Q17E) (Fig. 1B–D). Q17E slightly increased nuclear localization of wild-type PTEN (Fig. 1B,C). We confirmed similar effects of L42R on the localizations of wild-type PTEN, PTEN_A4_ and ePTEN in human embryonic kidney 293 (HEK293) cells (Fig. 1D).

To exclude the potential effects of C-terminal GFP on PTEN localization, we examined the localization of N-terminal GFP fusions and non-tagged versions of wild type PTEN, ePTEN, and nPTEN (Fig. S1). Both N-terminal fusions (Fig. S1A) and non-tagged versions (Fig. S1C) showed consistent intracellular distributions with C-terminal GFP fusions, indicating that PTEN localization is not affected by GFP. We noticed that the association of ePTEN with the plasma membrane was modestly affected by the chemical fixation for immunofluorescence (Fig. S1B and C). When cells were fixed, a portion of ePTEN appeared to dissociate from the plasma membrane. Therefore, it is critical to analyze PTEN localization using live cell imaging approaches to accurately determine its localization, especially at the plasma membrane.

To determine whether the L42R mutation changes the conformational state of PTEN_A4_ and ePTEN, we performed an “in trans” interaction assay; binding of GFP-tagged PTEN variants to a separate FLAG-tagged wild-type tail domain was assessed by co-immunoprecipitation using an anti-FLAG antibody. Wild-type PTEN-GFP did not interact with PTEN_352-403_-FLAG as it is in a closed conformation (Fig. 1E)^21^. In contrast, PTEN_A4_-GFP has an open conformation and showed a strong interaction with PTEN_352-403_-FLAG (Fig. 1E)^21^. Because ePTEN and nPTEN have mutations in the core region that block interactions with the C-terminal tail, they did not interact with PTEN_352-403_-FLAG even though they are maintained in an open conformation (Fig. 1E)^21^,^22^. Addition of the A4 mutations to ePTEN did not change its inability to interact with PTEN_352-403_-FLAG in ePTEN_A4_ (Fig. 1E). We found that L42R did not affect the interaction of PTEN_A4_-GFP or ePTEN-GFP with PTEN_352-403_-FLAG. PTEN_L42R,A4_ binds to PTEN_352-403_-FLAG while ePTEN_A4_ is defective in interactions with PTEN_352-403_-FLAG (Fig. 1E). Finally, we also found that L42R does not affect lipid phosphatase activities of PTEN_A4_ or ePTEN (Figs 1F and S2). Thus, PTEN with open conformations, whether mutations are located in the C-terminal tail (PTEN_A4_) or the core region (ePTEN), are recruited from the cytosol to the nucleus or the plasma membrane. Our data also show that dissociation of open conformation PTENs from the plasma membrane redistribute to the nucleus.

### The majority of PTEN_A4_ remains in the cytosol upon the breakdown of the nuclear membrane during mitosis

**Figure 2:**
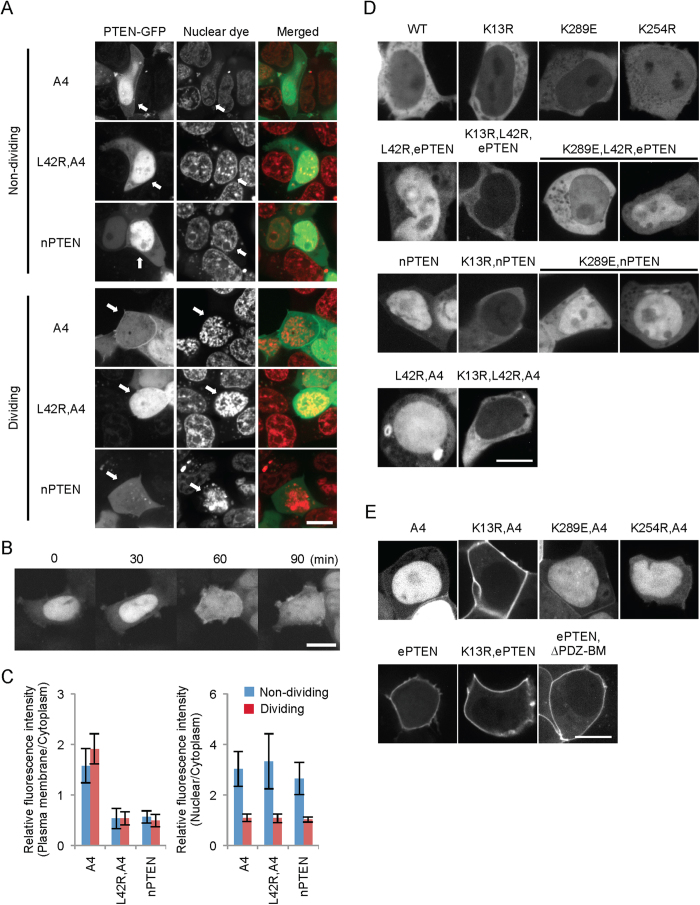
PTEN_A4_ is redistributed to the plasma membrane in the absence of the nuclear membrane during mitosis. (**A**) HEK293 cells expressing the indicated versions of PTEN-GFP were stained with a DNA dye, DRAQ5 (Cell Signaling), and observed by fluorescence microscopy. Dividing and non-dividing cells were identified by nuclear staining. Bar, 10 μm. (**B**) HEK293 cells expressing PTEN_A4_-GFP were observed by time-lapse confocal microscopy. PTEN_A4_-GFP was translocated to the plasma membrane upon nuclear membrane breakdown, which happened between 30 and 60 min. (**C**) Intensity of GFP at the plasma membrane and in the nucleus was quantified relative to that in the cytosol. Values represent the mean ± SD (n ≥ 8). (**D**,**E**) K13R blocks the nuclear accumulation of PTEN with the open conformation. HEK293 cells expressing the indicated forms of PTEN-GFP were observed by fluorescence microscopy. Bar, 10 μm.

Next, we wanted to know if PTEN_A4_ in the nucleus is recruited to the plasma membrane when released from the nucleus. To address this question, we examined PTEN_A4_-GFP in HEK293 cells at mitotic phases when the nuclear envelope is physiologically lost. In non-dividing cells, PTEN_A4_-GFP is associated with the plasma membrane and nucleus (Fig. 2A). To identify dividing cells, we examined the morphology of nuclear DNA using a fluorescent dye for DNA, DRAQ5. PTEN_A4_ was diffused into the cytosol and slightly increased the localization at the plasma membrane in dividing cells (Fig. 2A). Time-lapse fluorescence microscopy clearly showed that PTEN_A4_-GFP localized diffusely to the cytosol and to the plasma membrane upon breakdown of the nuclear envelope (Fig. 2B,C). Furthermore, when we examined the localization of PTEN_L42R,A4_-GFP and nPTEN-GFP in dividing cells, both were localized solely to the cytosol (Fig. 2A). It is appears that defects in membrane association of PTEN_L42R,A4_-GFP and nPTEN-GFP are not due to their sequestration in the nucleus.

It has been shown that ubiquitination and sumoylation of PTEN at lysine residues 13, 254 and 289 stimulates nuclear localization of PTEN^15^,^32^. However, when we substituted these lysines with arginine or glutamate in wild-type PTEN, we did not observe clear exclusion of PTEN_K13R_, PTEN_K254R_ or PTEN_K289E_ from the nucleus (Fig. 2D). We then used two PTEN variants, nPTEN and ePTEN_L42R_, which noticeably accumulate in the nucleus (Fig. 2D). While K289E modestly excluded nPTEN and ePTEN_L42R_ from the nucleus, K13R dramatically relocated nPTEN and ePTEN_L42R_ to the cytosol. We further introduced K13R into PTEN_L42R_,_A4_ and found similar exclusion from the nucleus. Therefore, K13, but not K289, plays a major role in the nuclear accumulation of open conformation PTENs. We then tested whether nuclear exclusion of open conformation PTENs stimulate redistribution to the plasma membrane (Fig. 2E). Importantly, when we combined the A4 and K13R mutations, PTEN_K13R,A4_ was dramatically recruited to the plasma membrane. In contrast, PTEN_K254R,A4_ and PTEN_K289E,A4_ showed distributions similar to PTEN_A4_. Overexpression of a deubiquitylating enzyme, USP7/HAUSP which regulates the nuclear localization of wild-type PTEN^33^, did not affect the nuclear localization of PTENA4 (Fig. S3). We observed no additional effects of K13R on the localization of ePTEN, which is fully associated with the plasma membrane. To further define the mechanism by which PTEN binds to the plasma membrane, we deleted its PDZ-binding domain, since this domain has been suggested to mediate association of PTEN with the plasma membrane through interactions with PDZ domain-containing membrane proteins such as MAGI2 and NHERF^34^,^35^. However, the truncation of the PDZ-binding domain did not affect membrane association of ePTEN (Fig. 2E).

**Figure 3:**
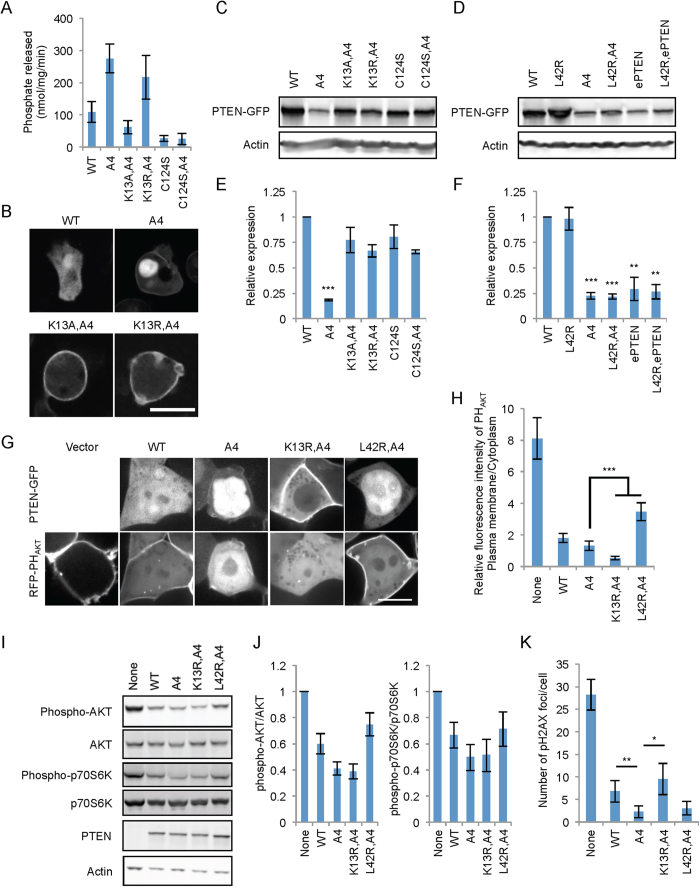
K13 controls the stability of PTEN, but not its membrane association. (**A**) The indicated PTEN-GFP proteins were immunopurified from *Dictyostelium* cells, and phosphatase activities were measured. Values represent the mean ± SD (n = 3). (**B**) *Dictyostelium* cells expressing various PTEN-GFP constructs were observed by fluorescence microscopy. Bar, 10 μm. (**C**,**D**) Immunoblotting of whole-cell lysates prepared from *Dictyostelium* cells expressing PTEN-GFP variants was performed using antibodies to GFP and actin. (**E**,**F**). The band intensity was quantified relative to actin. Values represent the mean ± SD (n = 3). (**G**) HEK293 cells expressing the PIP3 biosensor RFP-PH_AKT_ along with the indicated forms of PTEN-GFP were observed by fluorescence microscopy. Bar, 10 μm. (**H**) Intensity of RFP at the plasma membrane was quantified relative to that in the cytosol. Values represent the mean ± SD (n ≥ 8). (**I**) HEK293T cell lysates containing the indicated PTEN-GFP constructs were analyzed by immunoblotting with antibodies to phospho-AKT, AKT, phospho-p70S6K, p70S6K, PTEN, and actin. (**J**) Quantification of band intensity. The band intensity of phospho-AKT and phospho- p70S6K was quantified relative to AKT and p70S6K, respectively. Values represent the means ± SDs (n = 3). (**K**) Quantification of γH2AX foci after 16-h hydroxyurea treatment. Values represent the means ± SEMs (n = 10).

Since K13 is located in the PIP2-binding domain, this positively charged residue has also been suggested to be important for association of PTEN with the plasma membrane and therefore PTEN’s lipid phosphatase activity^28^,^36^. Consistent with this notion, we and others have previously shown that K13A inhibits lipid phosphatase activity of PTEN_A4_ (Fig. 3A)^22^,^36^,^37^. By contrast, when we substituted K13 with arginine (K13R), the lipid phosphatase activity was not affected in PTEN_K13R,A4_ (Fig. 3A). Importantly, neither K13A nor K13R decreased association of PTEN_A4_ with the plasma membrane (Fig. 3B). These data suggest that a positive residue at amino acid position 13 is critical for the enzymatic activity of PTEN, but not for its membrane association. Therefore, decreased enzymatic activity in PTEN_K13A,A4_ are not due to its dissociation from the membrane.

### Stability of open conformation PTEN depends on K13

In addition to subcellular localization, the conformation status of PTEN appears to regulate its stability. PTEN_A4_ and ePTEN have decreased protein stability compared to wild-type PTEN (Fig. 3C–F). Decreased stability seems to be due to proteasome-mediated protein degradation, as the stability of PTEN_A4_ and ePTEN was improved by the proteosomal inhibitor MG132^23^. Since K13 is subject to ubiquitination^14^, we tested the effect of K13A or K13R on PTEN_A4_ stability. Steady state amounts of PTEN_K13A,A4_ and PTEN_K13R,A4_ were higher than that of PTEN_A4_ (Fig. 3C,D). Therefore, K13 likely regulates the stability of open conformation PTENs. Previous studies have suggested that the loss of the lipid phosphatase activity stabilizes PTEN_A4_, but the underlying mechanisms for the increased stability of PTEN_K13R,A4_ observed here are independent of the loss of the enzymatic activity, as PTEN_K13R,A4_ maintained normal activity (Fig. 3A).

Furthermore, we have previously proposed that decreased stability of PTEN_A4_ and ePTEN results from increased association with the plasma membrane^22^. To test this model, we introduced the membrane dissociating substitution L42R to PTEN_A4_ and ePTEN. L42R did not increase levels of PTEN_A4_ or ePTEN (Fig. 3E,F) even though PTEN_L42R,A4_ and ePTEN_L42R_ were dissociated from the plasma membrane (Fig. 2D). Therefore, our data suggest that open conformations, rather than membrane association, decrease the stability of PTEN.

### K13R potentiates the function of PTEN_A4_ in suppression of PIP3 signaling

To examine the functional impact of increased membrane recruitment of PTEN_K13R,A4_ on PIP3 signaling, we co-expressed the PIP3 biosensor RFP-PH_AKT_ (RFP fused to the PIP3 binding pleckstrin homology (PH) domain of AKT^30^) in cells expressing PTEN_K13R,A4_ (Fig. 3G). RFP-PH_AKT_ was associated with the plasma membrane in HEK293 cells. PTEN_K13R,A4_ showed stronger effects on dissociation of RFP-PH_AKT_ from the plasma membrane compared to PTEN_A4_ or wild-type PTEN. A negative control, PTEN_L42R,A4_ which is defective in association with the plasma membrane, did not decrease levels of RFP-PH_AKT_ at the plasma membrane (Fig. 3G,H). To further assess PIP3 signaling, we examined the status of AKT and p70S6K, which are phosphorylated downstream of PI3K signaling (Fig. 3I,J). PTEN_A4_ and PTEN_A4,K13R_ more strongly suppressed these phosphorylation events compared to the wild-type PTEN. In contrast, the dissociation of PTEN_A4_ from the plasma membrane mediated by L42R decreased the ability of PTEN_A4_ to suppress the phosphorylation of its downstream substrates. These results show that increasing the membrane association of PTEN_A4_ by adding K13R further strengthened the function of PTEN_A4_ as a negative regulator of PIP3 signaling.

Recent studies have shown that PTEN functions in nuclear DNA repair^14–16^. We tested whether its nuclear localization is important for this function. The number of phosphorylated histone H2AX (γH2AX) foci has been used as a readout of DNA damage^16^,^38^. We treated HCT116 PTEN-null cells expressing PTEN-GFP, PTEN_A4_-GFP, PTEN_K13R,A4_-GFP, or PTEN_L42R,A4_-GFP with 1 mM hydroxyurea for 16 hours and then analyzed γH2AX foci using immunofluorescence microscopy. We found that PTEN_A4_-GFP significantly decreased the number of γH2AX foci compared to wild-type PTEN-GFP (Fig. 3K). Importantly, the K13R substitution, which blocks PTEN_A4_-GFP nuclear accumulation, decreased this function of PTEN_A4_-GFP. This was not observed in cells with the K42R substitution, which blocks the association of PTEN_A4_-GFP with the plasma membrane (Fig. 3K).

### NEDD4-1 preferentially binds to PTEN in open conformations and controls PTEN stability, but not PTEN localization

**Figure 4:**
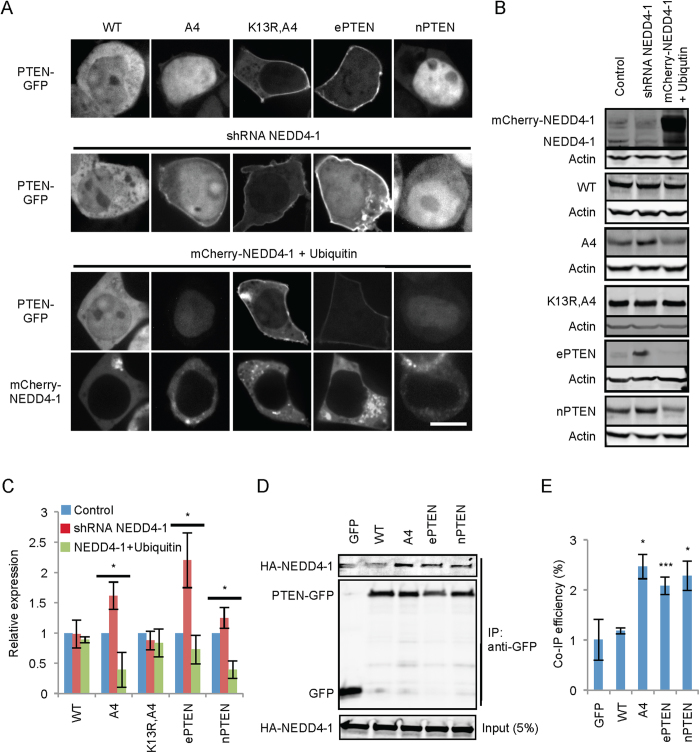
NEDD4-1 is required for the stability of PTEN, but not PTEN localization. (**A**) NEDD4-1 was knocked down using shRNAs in HEK293 cells expressing the indicated forms of PTEN-GFP (middle images). mCherry-NEDD4-1 was overexpressed together with HA-ubiquitin in the same set of cells (bottom panels). Cells were observed by fluorescence microscopy. Bar, 10 μm. (**B**) Whole-cell lysates were analyzed using immunoblotting with antibodies to NEDD4-1, actin and GFP to detect the indicated PTEN-GFP fusions. (**C**) Band intensity was quantified. Values represent the mean ± SD (n = 3). (**D**) Co-immunoprecipitation of NEDD4-1 and PTEN variants. HEK293 cells expressing HA-NEDD4-1 along with the indicated forms of PTEN-GFP were lysed and incubated with anti-GFP antibodies. Cell lysates (input) and immunoprecipitated fractions (IP) were analyzed by immunoblotting with antibodies to HA and GFP. (**E**) Band intensity was quantified. Values represent the mean ± SD (n = 3).

An E3 ubiquitin ligase, NEDD4-1, has been suggested to mediate ubiquitination of PTEN^15^. To test whether nuclear localization of PTEN_A4_ and nPTEN depends on NEDD4-1, we knocked down NEDD4-1 expression using shRNAs in HEK293 cells expressing PTEN_A4_-GFP or nPTEN-GFP. We found that unlike the K13R substitution, NEDD4-1 knockdown did not entirely exclude PTEN_A4_-GFP and nPTEN-GFP from the nucleus (Fig. 4A). We observed increased membrane association of PTEN_A4_-GFP when NEDD4-1 was knocked down, perhaps due to the increased PTEN_A4_-GFP levels. We also overexpressed mCherry-NEDD4-1 along with HA-ubiquitin. mCherry-NEDD4-1 was mainly localized in the cytosol and did not affect subcellular localization of wild-type PTEN, PTEN_A4_, PTEN_K13R,A4_, ePTEN or nPTEN (Fig. 4A).

We also tested whether NEDD4-1 is involved in decreased stability of PTEN_A4_, ePTEN and nPTEN. shRNA knockdown of NEDD4-1 increased the levels of all the PTEN variants, but not the levels of wild-type PTEN. Furthermore, overexpression of NEDD4-1 decreased the amounts of PTEN_A4_, ePTEN and nPTEN, but not of wild-type PTEN. PTEN_K13R,A4_ blocked the effect of knockdown and overexpression of NEDD4-1 on PTEN stability, suggesting that K13 is a key site for ubiquitination mediated by NEDD4-1, (Fig. 4A–C). These data suggest that open conformation PTENs are ubiquitinated at K13 by NEDD4-1, causing decreased protein stability. To understand how open conformation PTENs are regulated by NEDD4-1, we examined interactions of NEDD4-1 with different PTEN variants using co-immunoprecipitation (Fig. 4D,E). NEDD4-1 preferentially bound to PTEN_A4_-GFP, ePTEN-GFP and nPTEN-GFP with approximately 2.5 increases compared to GFP alone and wild-type PTEN-GFP. These increased interactions are likely the molecular basis for the specific effect of NEDD4-1 on the stability of PTEN_A4_, ePTEN and nPTEN.

### Opening the conformation of PTEN facilitates its import into the nucleus

**Figure 5:**
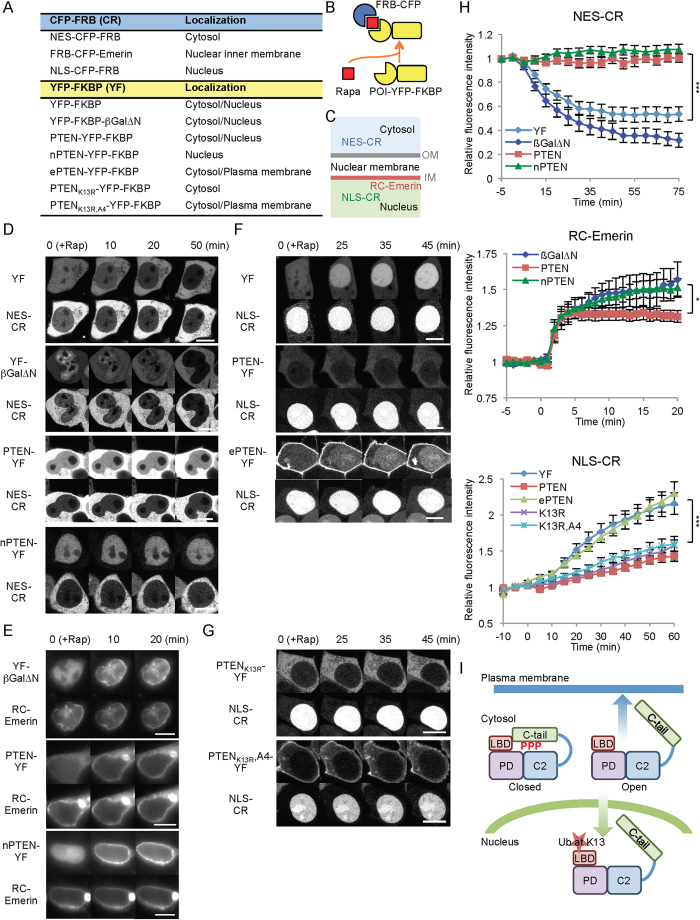
PTEN in the open conformation states, but not in closed states, is imported into the nucleus. (**A**) List of constructs used. (**B**) Experimental design. FRB and FKBP dimerize in the presence of rapamycin. (**C**) Recruiters (FRB fusions) are targeted to the cytosol (NES-CR), the nucleus (NLS-CR) or the nuclear inner membrane (RC-Emerin). Bar, 10 μm. (**D**) Rapamycin was added to HEK293 cells expressing NES-CR along with different recruitees (FKBP fusions). (**E**) Rapamycin was added to HEK293 cells expressing RC-emerin along with different recruitees. (**F**,**G**) Rapamycin was added to HEK293 cells expressing NLS-CR along with different recruitees. The fluorescence intensity in (**D**–**G**) was quantified in (**H**). Values represent the mean ± SEM (n ≥ 5). (**I**) A model. Opening the conformation of PTEN regulates its recruitment to the plasma membrane and nucleus.

To define the mechanisms of nuclear transport of PTEN in open conformation states, we developed a trap assay using the chemically inducible dimerization system, in which the FK506-binding protein (FKBP) and the rapamycin-binding domain of mTOR (FRB) stably dimerize in the presence of rapamycin (Fig. 5A,B)^39^. First, we fused FRB to CFP carrying nuclear exclusion signal (NES), NES-CFP-FRB. As a control, NES-CFP-FRB was co-expressed with YFP-FKBP. In the absence of rapamyin, NES-CFP-FRB is predominantly localized to the cytosol, while YFP-FKBP is present in both the cytosol and nucleus (Fig. 5C). Upon addition of rapamycin, YFP-FKBP was redistributed from the nucleus to the cytosol within 30 min (Fig. 5D,H). NES-CFP-FRB remained in the cytosol after the rapamycin addition. These results suggest that YFP-FKBP shuttles between the cytosol and nucleus and is trapped by NES-CFP-FRB in the cytosol. To determine the effect of protein sizes, we fused YFP-FKBP to a truncated version of beta galactosidase (~150 kDa) (YFP-βGal∆N-FKBP), which is larger than YFP (~25 kDa) and PTEN (~50 kDa). YFP-βGal∆N-FKBP showed kinetics of nuclear exclusion similar to that of YFP-FKBP, demonstrating that our assay is not affected by differences in protein size within this range (25–150 kDa). When we examined PTEN-YFP-FKBP, this protein was localized to both the cytosol and nucleus in the absence of rapamycin. However, in contrast to YFP-FKBP and YFP-βGal∆N-FKBP, the nuclear population of PTEN-YFP-FKBP persistently remained in the nucleus even after the addition of rapamycin (Fig. 5D,H). Similarly, nPTEN-YFP-FKBP was stably localized in the nucleus.

We hypothesized that the persistent localization of PTEN-YFP-FKBP and nPTEN-YFP-FKBP in the nucleus can be explained by the following two models. In the first model, PTEN-YFP-FKBP and nPTEN-YFP-FKBP are stably associated with intra nuclear structures and therefore immobile in the nucleus. In the second model, these proteins are freely diffusible in the nucleus but not exported out of the nucleus. To differentiate between these models, we examined the ability of PTEN-YFP-FKBP and nPTEN-YFP-FKBP to diffuse within the nucleus and their ability to be translocated to the inner nuclear membrane. We fused CFP-FRB to an inner nuclear membrane protein, emerin (Fig. 5C). Before the addition of rapamycin, both PTEN-YFP-FKBP and nPTEN-YFP-FKBP were relatively uniformly distributed in the nucleus. After the addition of rapamycin, both proteins were rapidly recruited to the inner surface of the nucleus within ~2 min, similar to the control protein YFP-βGal∆N-FKBP (Fig. 5E,H). Therefore, PTEN-YFP-FKBP and nPTEN-YFP-FKBP are diffusible within the nucleus. Our data support the model that PTEN-YFP-FKBP and nPTEN-YFP-FKBP are freely mobile inside the nucleus but not exported from the nucleus within the time range examined.

During the course of this experiment, we noticed that amounts of YFP-βGal∆N-FKBP and nPTEN-YFP-FKBP significantly increased on the inner nuclear surface within ~20 min of the addition of rapamycin. Conversely, such increases were not seen with PTEN-YFP-FKBP (Fig. 5H). In both cases, FRB-CFP-emerin remained in the nucleus after the rapamycin addition (Fig. 5E). These observations suggest that PTEN in open conformation states is continuously imported into the nucleus, while PTEN in a closed conformation state is not. To test this model, we fused CFP-FRB to a nuclear localization signal, NLS-CFP-FRB (Fig. 5C). We co-expressed NLS-CFP-FRB along with PTEN-YFP-FKBP, ePTEN-YFP-FKBP or YFP-FKBP (Fig. 5F,H). We used ePTEN because the majority of ePTEN is not in the nucleus, increasing the sensitivity of detection of its translocation into the nucleus. Upon addition of rapamycin, we observed similar dynamics of nuclear accumulation of ePTEN-YFP-FKBP and YFP-FKBP. In contrast, the time course of nuclear import of PTEN-YFP-FKBP was significantly slower (Fig. 5F,H). Furthermore, K13R blocked nuclear import of PTEN_A4_ (Fig. 5G,H). This import defect likely underlies defects in nuclear localization of PTEN_K13R,A4_. Finally, we examined the effect of the nuclear export inhibitor leptomycin B. While PTEN_A4_ remained in the nucleus in the presence of leptomycin B, nuclear PTEN_A4,K13R_ did not accumulate (Fig. S4), further supporting our hypothesis that the K13R mutation blocks the nuclear import of PTEN_A4_.

### Effects of PTEN-PTEN interactions on PTEN localization

**Figure 6:**
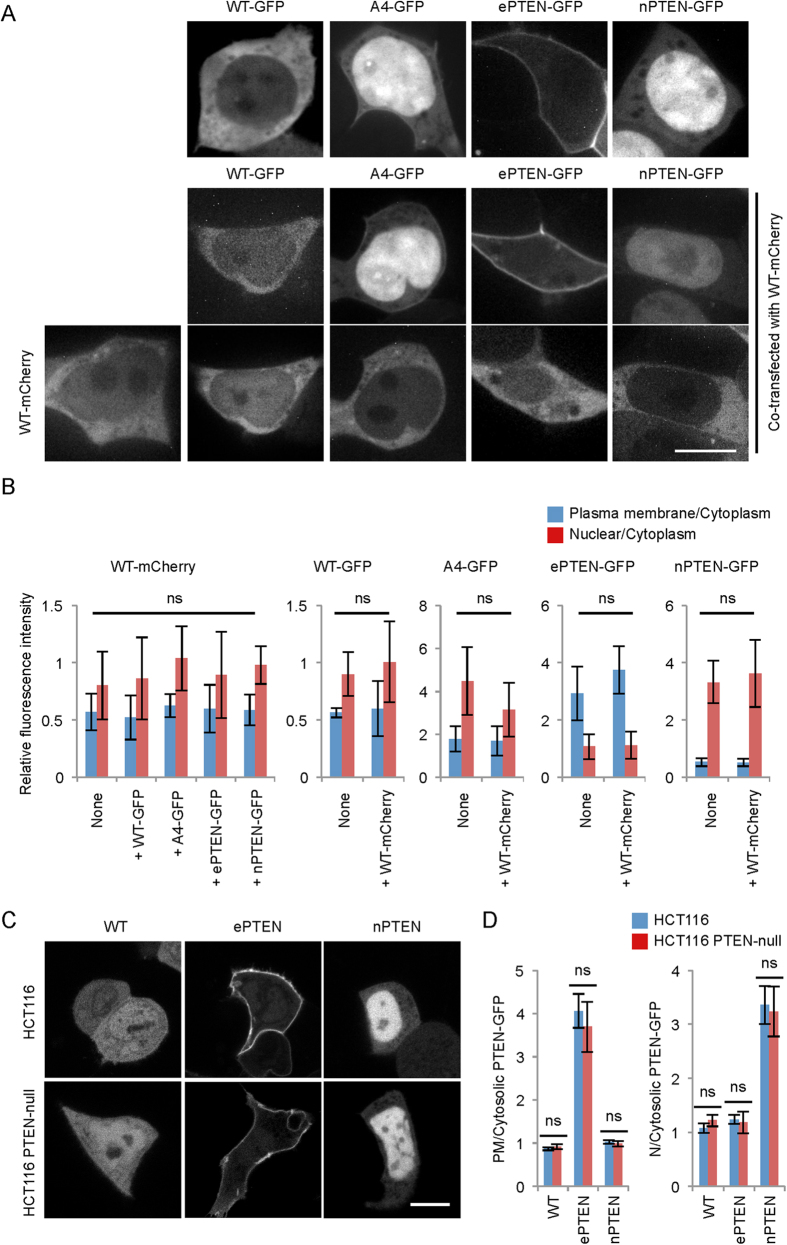
Effects of PTEN-PTEN interactions on PTEN localization. (**A**) Fluorescent microscopy images of HEK293 cells expressing wild-type PTEN-mCherry and different PTEN-GFP variants. Bar, 10 μm. (**B**) The intensities of mCherry and GFP at the plasma membrane and in the nucleus were quantified relative to those in the cytosol. Values represent the means ± SDs (n = 8). (**C**) Fluorescent microscopy images of HCT116 and HCT116 PTEN-null cells carrying the indicated constructs. Bar, 10 μm. (**D**) Intensity of GFP at the plasma membrane and in the nucleus was quantified relative to that in the cytosol. Values represent the mean ± SEM (n ≥ 8).

PTEN forms a homodimer, and this protein-protein interaction is promoted when the phosphorylation of the C-terminal tail is blocked and the conformation is opened^40^. To test whether the intracellular distribution of PTEN variants is affected by PTEN-PTEN interactions, we co-expressed mCherry tagged wild-type PTEN with GFP-tagged wild-type PTEN, PTEN_A4_, ePTEN, or nPTEN in HEK293 cells (Fig. 6A,B). We found no effects of these combinations on PTEN localization. Furthermore, we compared the localization of wild-type PTEN-GFP, ePTEN-GFP, and nPTEN-GFP in HCT116 cells and PTEN-null HCT116 cells (Fig. 6C,D). We found that these PTEN variants were similarly distributed in these two cell types, suggesting that the effect of endogenous PTEN is negligible.

## Discussion

In this report, we show that open conformation states of PTEN are required for the localization of this protein to the nucleus and plasma membrane (Fig. 5I). The majority of PTEN exists in a closed conformation that is maintained by the association of its C-terminal tail with the core region on the surface of PTEN molecules. This intramolecular interaction is controlled by phosphorylation of serine/threonine clusters in the C-terminal region. Previous studies have identified casein kinase II as a key regulatory kinase that phosphorylates the PTEN serine/threonine cluster^41^. When the tail region is phosphorylated, the intramolecular interactions that maintain the closed conformation are promoted. In contrast, when the tail becomes dephosphorylated, it dissociates from the core region, opening the conformation of PTEN. This conformational change exposes the membrane-binding regulatory interface and stimulates the recruitment of PTEN to the plasma membrane. In addition, opening the conformation also makes K13 accessible to E3 ubiquitin ligases and promotes the transport of PTEN to the nucleus, presumably through ubiquitination. A recent study using bioluminescence resonance energy transfer has further shown the importance of the phosphorylation-mediated conformational change of PTEN upon activation of G protein coupled receptors^42^. Our current findings and previous studies suggest that the conformational change of PTEN is a critical mechanism that regulates the functional localization of PTEN. In the crystal structure of PTEN, the unstructured tail region was not included^25^. It would be important to elucidate how the tail and core region interact at the structural level in future studies.

Our data also suggest a dynamic equilibrium between membrane localization and nuclear localization of open conformation PTENs. Since PTEN_A4_, which is trapped in an open conformation because of the inhibition of its tail phosphorylation, is predominantly accumulated in the nucleus, there appears to be stronger PTEN nuclear import activity than recruitment to the plasma membrane. This relatively high nuclear import activity might function to sequester open conformation PTENs away from the plasma membrane to maintain required levels of PIP3. Previous studies have shown that nuclear localization of PTEN changes in different physiological contexts. For example, levels of nuclear PTEN increase in quiescent cells and decrease in proliferating cells. Similarly, cancer cells have decreased amounts of nuclear PTEN. These changes in the abundance of nuclear PTEN may reflect the regulation of conformations of PTEN in these cells. Moreover, various effects of substitution of lysines 13 and 289 have been reported in previous studies. As closed conformations of PTEN are not regulated by these lysine substitutions, the reported differences may result from differences in the levels of PTEN with open conformations.

The PIP2 binding motif consists of a cluster of five positively charged lysine and arginine residues in the N-terminal region of PTEN. Similar PIP2 binding motifs have been found and characterized in several actin binding proteins, such as gelsolin and WASP^43^. Positively charged residues help PTEN interact with negatively charged phospholipids including PIP2, PIP3 and phosphatidylserine in the plasma membrane as truncation of the PIP2 binding motif blocks association of PTEN with the plasma membrane. It has been suggested that K13 in the PIP2 binding motif is important for enzymatic activity because it recruits PTEN to the plasma membrane, and its substitution to alanine blocks activity^22^,^28^,^36^,^37^. However, we found that K13A increased the recruitment of PTEN_A4_ to the plasma membrane. Therefore, the role of K13 in the enzymatic activity is not due to the association of PTEN with the plasma membrane. Since substitution of lysine to arginine does not decrease PTEN enzymatic activity but increases PTEN-membrane association, positive charges at amino acid position 13 are important for the enzymatic activity of PTEN and its enzymatic role is separate from its association with plasma membrane.

PTEN localization studies have been hampered by the fact that the majority of wild-type PTEN is phosphorylated and present in closed conformations. In this study, we genetically engineered PTEN to maintain the protein in an open conformation and thus bypassed the inhibitory mechanism that keeps PTEN in the cytosol. Experimental manipulation of PTEN conformations allowed us to define the mechanisms that drive PTEN to the plasma membrane and nucleus. Cancer-associated mutations in PTEN cause changes throughout the protein’s structure. Based on our findings, demonstrating the importance of the open conformation of PTEN for its localization and activity, we predict that there are cancer-related mutations that render a open PTEN conformation. These mutations may affect the linker between the core and tail regions; this part of the protein has not been included in the crystal structure of PTEN and is likely important for stabilizing interactions between the core and tail regions independently of tail phosphorylation. We are currently searching for such mutations in the pool of oncogenic PTEN mutations. In future studies, it will be important to characterize the effects of such mutations on PTEN localization, stability, and activity, which may lead to the development of cancer interventions based on the activation of PTEN’s tumor suppressor function.

## Methods

### Cells and plasmids

*Dictyostelium* cells were cultured in HL5 medium at 22 °C^23^. Cells expressing GFP fused to PTEN variants were selected in the presence of 20 μg/ml G418 for more than 2 weeks. HEK293 cells were grown in DMEM containing 10% FBS. Cells were transiently transfected with 1 μg of DNA plasmids on eight-well chambered coverglass (Lab-TekII, Nunc) using Lipofectamine 3000 (Life Technologies), according to the manufacturer’s protocol. Transfected cells were cultured overnight, then analyzed. The plasmids used in this study are listed in Table S1. PTEN variants were generated using overlap extension PCR^44^ and cloned into pKF3, a *Dictyostelium* expressing plasmid carrying GFP^23^,^30^, and pcDNA3.1, a mammalian expression vector (Life Technologies). All constructs were confirmed by DNA sequencing.

### Fluorescence microscopy

*Dictyostelium* cells expressing PTEN-GFP constructs were resuspended in DB containing 20 μM MG132 to block proteosomal degradation of PTEN-GFP and placed on eight-well chambered coverglass (Lab-TekII, Nunc), as previously described^23^,^30^. To analyze PTEN-GFP in HEK293 cells, the culture medium was replaced with Leibovitz’s L-15 medium (Life Technologies) before observations. Fluorescent images were obtained using a Leica DMI 6000 inverted microscope equipped with a 63x objective and a CoolSNAP EZ camera. Time-lapse images of cell division that were obtained using a 40x objective on a 3i Marianis/Yokogawa Spinning Disk Confocal microscope equipped with a CO_2_, humidity and temperature-controlled enclosure. All images were analyzed using Image J software. To determine fluorescence intensity of PTEN-GFP at the plasma membrane and nucleus relative to the cytosol, we averaged fluorescence intensity in a 1 pixel area from three different positions in each subcellular compartment. We subtracted background fluorescence intensity from each measurement. The position of the nucleus was determined by DAPI staining.

### Immunofluorescence

Cells were fixed using PBS containing 4% paraformaldehyde. Fixed cells were washed in PBS, permeabilized with 0.1% Triton X-100/PBS, and blocked in 0.5% BSA/PBS. The cells were incubated with antibodies to PTEN (138G6, Cell Signaling), followed by the appropriate secondary antibodies. Samples were viewed using a Zeiss LSM510-Meta laser scanning confocal microscope.

### Interactions between PTEN and its C-terminal tail

Association of PTEN and its C-terminal tail (PTEN_352-403_-YFP-FLAG) was analyzed, as previously described^21^,^30^. HEK293 cells were transiently transfected with a plasmid carrying PTEN_352-403_-YFP-FLAG and then cultured overnight. Cells were lysed in lysis buffer containing 1% Nonidet P-40, 50 mM NaCl, 20 mM Tris-HCl (pH 7.5), 10% glycerol, 0.1 mM EDTA, phosphatase inhibitor cocktail (Sigma) and protease inhibitor cocktail (Roche). The lysates were then cleared by centrifugation at 13,000 rpm for 20 min at 4 °C. *Dictyostelium* cells expressing GFP fused to wild-type and mutant PTEN were lysed in 1% Nonidet P-40, 300 mM NaCl, 10 mM Tris-HCl (pH 7.5), 2 mM EDTA, phosphatase inhibitor cocktail (Sigma) and protease inhibitor cocktail (Roche). The lysates were then cleared by centrifugation at 13,000 rpm for 20 min at 4 °C. 100 μl of the HEK293 cell lysates were mixed with 500 μl of the *Dictyostelium* cell lysates. 15 μl of beads coupled to anti-FLAG antibodies (Sigma) were added to the mixtures and incubated for two hours. The bound fractions were analyzed by SDS-PAGE and immunoblotting using antibodies to GFP, which recognize both GFP and YFP.

### Immunoblotting

Proteins were separated by SDS-PAGE and transferred onto PVDF membranes. Antibodies used against PTEN were: (138G6, Cell Signaling), GFP^45^, NEDD4-1 (ab14592, Abcam), p70S6K and phospho-p70S6k duet (8209, Cell Signaling), AKT (9272, Cell Signaling), phospho-AKT (4060, Cell Signaling) and actin (C-11, Santa Cruz Biotechnology). Immunocomplexes were visualized by fluorescently labeled secondary antibodies and detected using a PharosFX Plus molecular imager (Bio-Rad).

### Phosphatase activity

The phosphatase activity of PTEN was measured, as described previously^30^,^37^. Wild-type and mutant forms of PTEN fused to GFP were expressed in *Dictyostelium* cells and immunopurified using GFP-Trap agarose beads (ChromoTek). The enzymatic activity was determined by measuring release of phosphates from PIP3 diC8 using a Malachite Green Phosphatase assay kit (Echelon). The activity was normalized relative to amounts of purified PTEN-GFP proteins.

### Immunoprecipitation

Whole-cell lysates of HEK293 cells expressing different PTEN-GFP constructs were prepared as described above for “in trans” interaction assays. Beads conjugated with anti-GFP antibodies were added to the lysates and incubated for two hours. The bead-bound PTEN-GFP constructs were concentrated by removal of supernatant after centrifugation. 400 μl of clean whole-cell lysate of HEK293 expressing HA-NEDD4-1 were then added to the beads and incubated for three hours. The beads were washed three times, and the bound fractions were analyzed by SDS-PAGE and immunoblotting using antibodies to GFP and HA.

### DNA repair assays

HCT116 PTEN-null cells were seeded on Lab-Tek eight-well chamber coated with laminin (L2020, Sigma) and incubated overnight. Cells were treated with 1 mM hydroxyurea (H8627, Sigma) for 16 h after a 24-h transfection with either wild-type PTEN or mutated constructs. Cells were fixed as previously described^16^ and immunolabeled with a rabbit anti-phosphorylated histone H2AX (γH2AX) antibody (ab2893, Abcam) and a anti-rabbit IgG Alexa 568 antibody (Invitrogen). Images were acquired by confocal fluorescence microscopy (Zeiss LSM 780 NLO) equipped with a 63× objective. The numbers of nuclear foci of phosphorylated histone H2AX were manually counted. Nuclei were identified by staining with Hoechst 33258 (Molecular Probes).

### shRNA

NEDD4-1 was knocked down in HEK293T cells by co-transfecting plasmids expressing shRNA1 (5′-GCTGAACTATACGGTTCAAAT-3′) and shRNA2 (5′-CGGTTGGAGAATGTAGCAATA-3′). After 24 h of transfection, transfectants were selected with 5–10 μg/ml of puromycin for at least one week. Efficiency of the knockdown was confirmed by immunoblotting using an antibody against NEDD4-1.

### Trap assays

HEK293 cells were transfected with DNA constructs by plating them directly in a transfection solution containing DNA plasmid and FuGENE HD (Roche). Cells were plated on poly-D-Lysine hydrobromide (Sigma)-coated cover glass, and then cultured in 6-well plates. Imaging was carried out 36 to 48 h after transfection. For imaging, DMEM medium containing 25 mM HEPES (Gibco) was used. Fluorescent images were obtained using either an epifluorescence microscope for translocation assay with emerin, or a confocal microscope for other assays. Using the epifluorescence microscope, CFP and YFP excitation were carried out by an X-Cite Series 120Q mercury vapor lamp that is processed through appropriate filter cubes. Images were taken using a 63× objective (Plan-Apochromat, Zeiss) mounted on an inverted Axiovert 135 TV microscope (Zeiss) and were captured by a QIClick charge-coupled device camera (QImaging). Imaging was driven by Metamorph 7.5 imaging software (Molecular Devices). For confocal microscopy, CFP and YFP excitation were carried out by lasers at 435 nm and 515 nm wavelength which are processed through appropriate filter cubes. Images were taken using a 63× objective with 1.4NA (Zeiss) mounted on an inverted microscope Cell Observer (Zeiss), and were captured by LSM780 scanner (Zeiss). Imaging was driven by Zen Black (Zeiss). All imaging experiments were carried out at room temperature (21–23 °C). For time-lapse imaging, fluorescence images were taken every 1 or 5 minutes for 25 to 80 min as indicated. Images were normally collected for 5 to 10 min before the addition of 100 nM rapamycin (0 minute). CFP intensity was normalized to the mean of the readings of the first two to three time points collected before the addition of rapamycin.

### Statistics

P values were calculated using the Student’s t-test: *p < 0.05; **p < 0.01; ***p < 0.001.

## Additional Information

**How to cite this article**: Nguyen, H.-N. *et al.* Opening the conformation is a master switch for the dual localization and phosphatase activity of PTEN. *Sci. Rep.* **5**, 12600; doi: 10.1038/srep12600 (2015).

## Supplementary Material

Supplementary Information

## References

[b1] Vanhaesebroeck B., Stephens L. & Hawkins P. PI3K signalling: the path to discovery and understanding. Nature reviews Molecular cell biology 13, 195–203 (2012). 10.1038/nrm3290 22358332

[b2] Sulis M. L. & Parsons R. PTEN: from pathology to biology. Trends in cell biology 13, 478–483 (2003). 12946627 10.1016/s0962-8924(03)00175-2

[b3] Manning B. D. & Cantley L. C. AKT/PKB signaling: navigating downstream. Cell 129, 1261–1274 (2007). 17604717 10.1016/j.cell.2007.06.009 PMC2756685

[b4] Worby C. A. & Dixon J. E. Pten. Annual review of biochemistry 83, 641–669 (2014). 10.1146/annurev-biochem-082411-113907 24905788

[b5] Shi Y., Paluch B. E., Wang X. & Jiang X. PTEN at a glance. Journal of cell science 125, 4687–4692 (2012). 23223894 10.1242/jcs.093765 PMC3517091

[b6] Chalhoub N. & Baker S. J. PTEN and the PI3-kinase pathway in cancer. Annu Rev Pathol 4, 127–150 (2009). 18767981 10.1146/annurev.pathol.4.110807.092311 PMC2710138

[b7] Ali I. U., Schriml L. M. & Dean M. Mutational spectra of PTEN/MMAC1 gene: a tumor suppressor with lipid phosphatase activity. J Natl Cancer Inst 91, 1922–1932 (1999). 10564676 10.1093/jnci/91.22.1922

[b8] Tan M. H., Mester J. L., Ngeow J., Rybicki L. A., Orloff M. S. & Eng C. Lifetime cancer risks in individuals with germline PTEN mutations. Clin Cancer Res 18, 400–407 (2012). 22252256 10.1158/1078-0432.CCR-11-2283 PMC3261579

[b9] Hollander M. C., Blumenthal G. M. & Dennis P. A. PTEN loss in the continuum of common cancers, rare syndromes and mouse models. Nat Rev Cancer 11, 289–301 (2011). 21430697 10.1038/nrc3037 PMC6946181

[b10] Song M. S., Salmena L. & Pandolfi P. P. The functions and regulation of the PTEN tumour suppressor. Nature reviews Molecular cell biology 13, 283–296 (2012). 22473468 10.1038/nrm3330

[b11] Leslie N. R., Batty I. H., Maccario H., Davidson L. & Downes C. P. Understanding PTEN regulation: PIP2, polarity and protein stability. Oncogene 27, 5464–5476 (2008). 18794881 10.1038/onc.2008.243

[b12] Carracedo A., Alimonti A. & Pandolfi P. P. PTEN level in tumor suppression: how much is too little? Cancer Res 71, 629–633 (2011). 21266353 10.1158/0008-5472.CAN-10-2488 PMC3249925

[b13] Nguyen H. N. *et al.* A New Class of Cancer-Associated PTEN Mutations Defined by Membrane Translocation Defects. Oncogene 10.1038/onc.2014.293. **In press**, (2014). PMC4377315 25263454

[b14] Song M. S. *et al.* Nuclear PTEN regulates the APC-CDH1 tumor-suppressive complex in a phosphatase-independent manner. Cell 144, 187–199 (2011). 21241890 10.1016/j.cell.2010.12.020 PMC3249980

[b15] Trotman L. C. *et al.* Ubiquitination regulates PTEN nuclear import and tumor suppression. Cell 128, 141–156 (2007). 17218261 10.1016/j.cell.2006.11.040 PMC1855245

[b16] Bassi C. *et al.* Nuclear PTEN controls DNA repair and sensitivity to genotoxic stress. Science 341, 395–399 (2013). 23888040 10.1126/science.1236188 PMC5087104

[b17] Lachyankar M. B. *et al.* A role for nuclear PTEN in neuronal differentiation. J Neurosci 20, 1404–1413 (2000). 10662831 10.1523/JNEUROSCI.20-04-01404.2000 PMC6772384

[b18] Planchon S. M., Waite K. A. & Eng C. The nuclear affairs of PTEN. Journal of cell science 121, 249–253 (2008). 18216329 10.1242/jcs.022459

[b19] Yu W., He X., Ni Y., Ngeow J. & Eng C. Cowden syndrome-associated germline SDHD variants alter PTEN nuclear translocation through SRC-induced PTEN oxidation. Hum Mol Genet 24, 142–153 (2015). 25149476 10.1093/hmg/ddu425 PMC4262496

[b20] Das S., Dixon J. E. & Cho W. Membrane-binding and activation mechanism of PTEN. Proceedings of the National Academy of Sciences of the United States of America 100, 7491–7496 (2003). 12808147 10.1073/pnas.0932835100 PMC164614

[b21] Rahdar M., Inoue T., Meyer T., Zhang J., Vazquez F. & Devreotes P. N. A phosphorylation-dependent intramolecular interaction regulates the membrane association and activity of the tumor suppressor PTEN. Proc Natl Acad Sci USA 106, 480–485 (2009). 19114656 10.1073/pnas.0811212106 PMC2626728

[b22] Nguyen H. N. *et al.* Engineering ePTEN, an enhanced PTEN with increased tumor suppressor activities. Proceedings of the National Academy of Sciences of the United States of America 111, E2684–E2693 (2014). 24979808 10.1073/pnas.1409433111 PMC4084459

[b23] Nguyen H. N., Afkari Y., Senoo H., Sesaki H., Devreotes P. N. & Iijima M. Mechanism of human PTEN localization revealed by heterologous expression in Dictyostelium. Oncogene 33, 5688–5696 (2014). 24292679 10.1038/onc.2013.507 PMC4041858

[b24] Gericke A., Leslie N. R., Losche M. & Ross A. H. PtdIns(4,5)P2-mediated cell signaling: emerging principles and PTEN as a paradigm for regulatory mechanism. Advances in experimental medicine and biology 991, 85–104 (2013). 23775692 10.1007/978-94-007-6331-9_6 PMC3763917

[b25] Lee J. O. *et al.* Crystal structure of the PTEN tumor suppressor: implications for its phosphoinositide phosphatase activity and membrane association. Cell 99, 323–334 (1999). 10555148 10.1016/s0092-8674(00)81663-3

[b26] Shenoy S. *et al.* Membrane association of the PTEN tumor suppressor: molecular details of the protein-membrane complex from SPR binding studies and neutron reflection. PLoS One 7, e32591 (2012). 22505997 10.1371/journal.pone.0032591 PMC3323581

[b27] Lumb C. N. & Sansom M. S. Defining the membrane-associated state of the PTEN tumor suppressor protein. Biophysical journal 104, 613–621 (2013). 23442912 10.1016/j.bpj.2012.12.002 PMC3566463

[b28] Walker S. M., Leslie N. R., Perera N. M., Batty I. H. & Downes C. P. The tumour-suppressor function of PTEN requires an N-terminal lipid-binding motif. The Biochemical journal 379, 301–307 (2004). 14711368 10.1042/BJ20031839 PMC1224073

[b29] Denning G., Jean-Joseph B., Prince C., Durden D. L. & Vogt P. K. A short N-terminal sequence of PTEN controls cytoplasmic localization and is required for suppression of cell growth. Oncogene 26, 3930–3940 (2007). 17213812 10.1038/sj.onc.1210175

[b30] Yang J. M., Nguyen H. N., Sesaki H., Devreotes P. N. & Iijima M. Engineering PTEN function: Membrane association and activity. Methods 77–78, 119–124 (2014). 10.1016/j.ymeth.2014.10.018 PMC4388803 25448479

[b31] Odriozola L., Singh G., Hoang T. & Chan A. M. Regulation of PTEN activity by its carboxyl-terminal autoinhibitory domain. The Journal of biological chemistry 282, 23306–23315 (2007). 17565999 10.1074/jbc.M611240200

[b32] Liu F., Wagner S., Campbell R. B., Nickerson J. A., Schiffer C. A. & Ross A. H. PTEN enters the nucleus by diffusion. Journal of cellular biochemistry 96, 221–234 (2005). 16088943 10.1002/jcb.20525

[b33] Song M. S. *et al.* The deubiquitinylation and localization of PTEN are regulated by a HAUSP-PML network. Nature 455, 813–817 (2008). 18716620 10.1038/nature07290 PMC3398484

[b34] Molina J. R., Morales F. C., Hayashi Y., Aldape K. D. & Georgescu M. M. Loss of PTEN binding adapter protein NHERF1 from plasma membrane in glioblastoma contributes to PTEN inactivation. Cancer Res 70, 6697–6703 (2010). 20736378 10.1158/0008-5472.CAN-10-1271 PMC2932801

[b35] Wu X. *et al.* Evidence for regulation of the PTEN tumor suppressor by a membrane-localized multi-PDZ domain containing scaffold protein MAGI-2. Proceedings of the National Academy of Sciences of the United States of America 97, 4233–4238 (2000). 10760291 10.1073/pnas.97.8.4233 PMC18208

[b36] Campbell R. B., Liu F. & Ross A. H. Allosteric activation of PTEN phosphatase by phosphatidylinositol 4,5-bisphosphate. The Journal of biological chemistry 278, 33617–33620 (2003). 12857747 10.1074/jbc.C300296200

[b37] Iijima M., Huang Y. E., Luo H. R., Vazquez F. & Devreotes P. N. Novel mechanism of PTEN regulation by its phosphatidylinositol 4,5-bisphosphate binding motif is critical for chemotaxis. J Biol Chem 279, 16606–16613 (2004). 14764604 10.1074/jbc.M312098200

[b38] Jadav R. S., Chanduri M. V., Sengupta S. & Bhandari R. Inositol pyrophosphate synthesis by inositol hexakisphosphate kinase 1 is required for homologous recombination repair. The Journal of biological chemistry 288, 3312–3321 (2013). 23255604 10.1074/jbc.M112.396556 PMC3561551

[b39] DeRose R., Miyamoto T. & Inoue T. Manipulating signaling at will: chemically-inducible dimerization (CID) techniques resolve problems in cell biology. Pflugers Arch 465, 409–417 (2013). 23299847 10.1007/s00424-012-1208-6 PMC3584178

[b40] Papa A. *et al.* Cancer-Associated PTEN Mutants Act in a Dominant-Negative Manner to Suppress PTEN Protein Function. Cell 157, 595–610 (2014). 24766807 10.1016/j.cell.2014.03.027 PMC4098792

[b41] Torres J. & Pulido R. The tumor suppressor PTEN is phosphorylated by the protein kinase CK2 at its C terminus. Implications for PTEN stability to proteasome-mediated degradation. The Journal of biological chemistry 276, 993–998 (2001). 11035045 10.1074/jbc.M009134200

[b42] Lima-Fernandes E. *et al.* A biosensor to monitor dynamic regulation and function of tumour suppressor PTEN in living cells. Nat Commun 5, 4431 (2014). 25028204 10.1038/ncomms5431

[b43] Yin H. L. & Janmey P. A. Phosphoinositide regulation of the actin cytoskeleton. Annual review of physiology 65, 761–789 (2003). 10.1146/annurev.physiol.65.092101.142517 12471164

[b44] Zhang P., Wang Y., Sesaki H. & Iijima M. Proteomic identification of phosphatidylinositol (3,4,5) triphosphate-binding proteins in Dictyostelium discoideum. Proc Natl Acad Sci USA 107, 11829–34118 (2010). 20547830 10.1073/pnas.1006153107 PMC2900710

[b45] Chen C. L., Wang Y., Sesaki H. & Iijima M. Myosin I links PIP3 signaling to remodeling of the actin cytoskeleton in chemotaxis. Science signaling 5, ra10 (2012). 22296834 10.1126/scisignal.2002446 PMC3381520

