# Supplementary material for: Opening the conformation is a master switch for the dual localization and phosphatase activity of PTEN
Source: Sci Rep. 2015 Jul 28;5:12600. doi: 10.1038/srep12600 (PMC4517176; doi:10.1038/srep12600)
Supplement: Supplementary Information [file srep12600-s1.pdf]

Supplementary Information for

Opening the conformation is a master switch for the dual localization and phosphatase activity of PTEN

Hoai-Nghia Nguyen, Jr-Ming Yang, Takafumi Miyamoto, Kie Itoh, Elmer Rho, Qiang Zhang, Takanari Inoue, Peter N. Devreotes, Hiromi Sesaki, and Miho Iijima*

Department of Cell Biology, The Johns Hopkins University School of Medicine, Baltimore, MD

*Correspondence: miijima@jhmi.edu

Figures S1-4
Tables S1 and S2

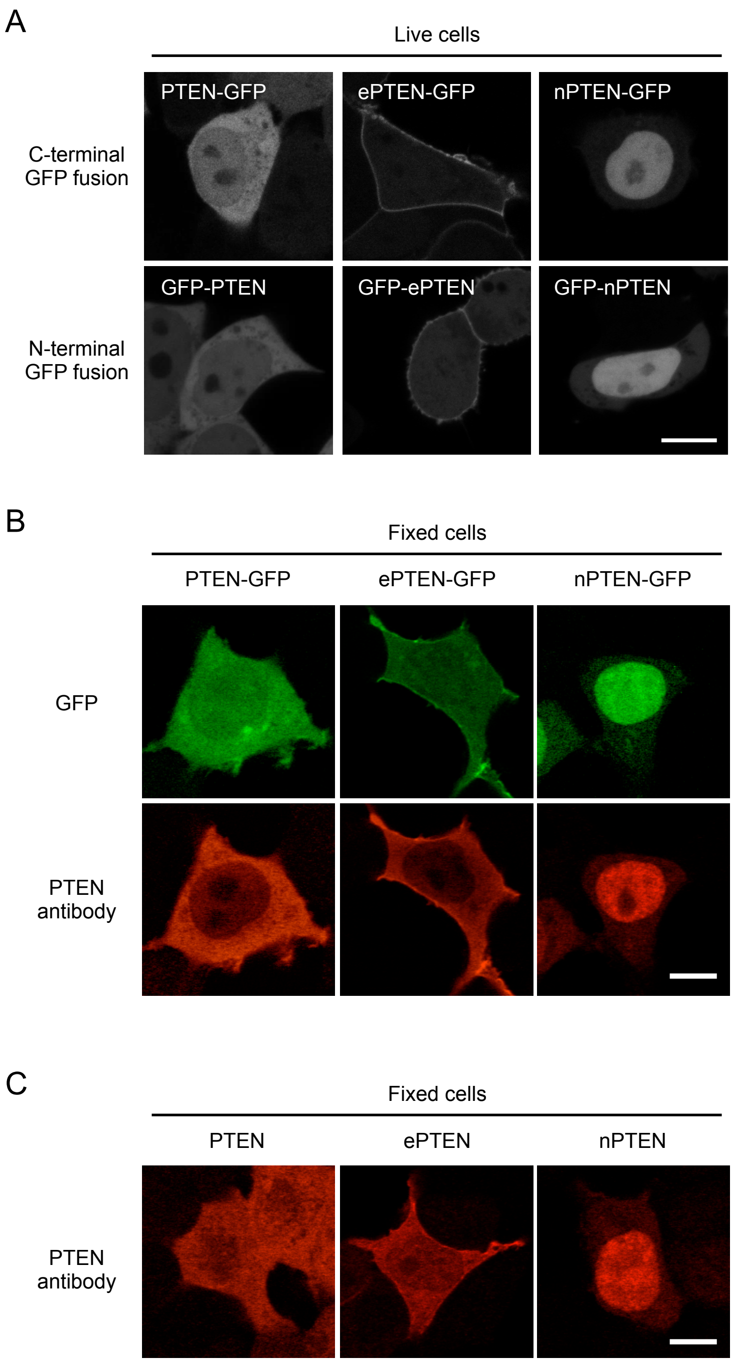

Figure S1. GFP does not affect PTEN localization. (A) Fluorescent microscopy images of HEK293 cells expressing the indicated PTEN-GFP (C-terminal GFP fusion) variants and GFP-PTEN (N-terminal GFP fusion) variants. Bar, 10 μm. (B) HEK293 cells expressing the indicated PTEN-GFP were fixed and processed using an anti-PTEN antibody. (C) HEK293 cells expressing the indicated non-tagged PTEN variants labeled with an anti-PTEN antibody.

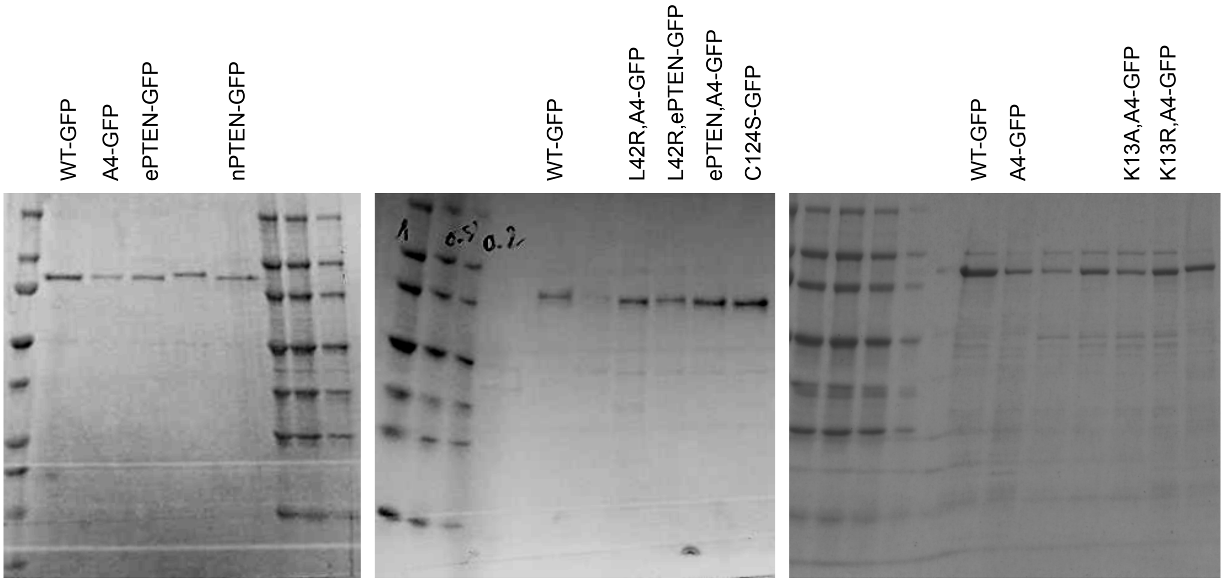

Purified proteins	μg/ml
WT	45.2
A4	18.2
ePTEN	23.5
nPTEN	30.7

Purified proteins	μg/ml
WT	40.8
L42R,A4	28.6
L42R,ePTEN	27.3
ePTEN,A4	39.3
C124S	44.9

Purified proteins	μg/ml
WT	65.1
A4	19.7
K13A,A4	24.2
K13R,A4	34.8

Figure S2. Immunopurification of PTEN-GFP variants. Purified proteins were analyzed by SDS-PAGE and Coomassie Brilliant Blue staining. Protein standards (Perfect protein marker, Millipore) were used to determine the concentrations of purified proteins.

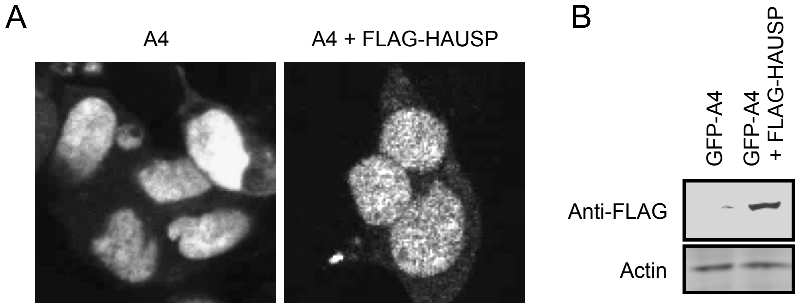

Figure S3. Effects of USP7/ HAUSP overexpression on PTEN localization. (A) USP7/HAUSP was overexpressed in HEK293 cells carrying $PTEN_{A4}$. (B) Immunoblotting of whole cell lysates from HEK293 cells expressing USP7/ HAUSP. Actin was used as a loading control.

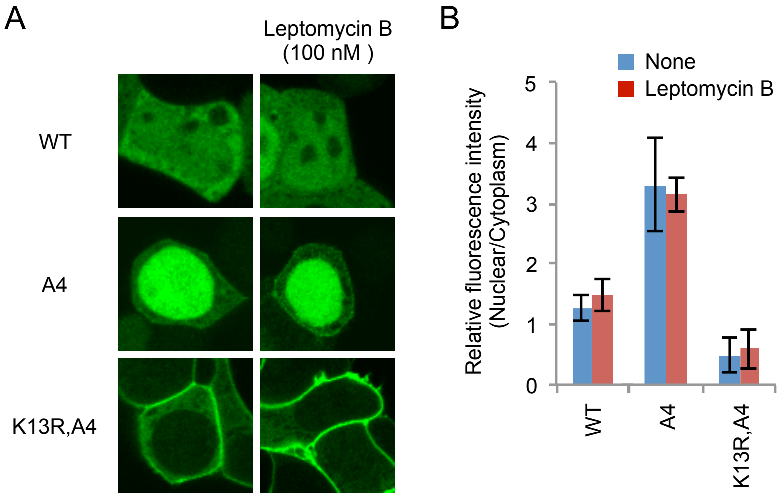

Figure S4. Effects of the nuclear export inhibitor leptomycin B on PTEN localization. After a 6-hour treatment with 100 nM leptomycin B, HEK293 cells expressing the indicated PTEN-GFP (C-terminal GFP fusion) variants and GFP-PTEN (N-terminal GFP fusion) variants were observed by fluorescence microscopy. Bar, 10 µm. (B) The intensity of GFP in the nucleus was quantified relative to that in the cytosol. Values represent the means ± SDs (n = 8).

Table S1. Plasmids used in this study

Plasmids	Description	References
pKF3		Kindly provided by Swaney K
pKF3-hPTEN		Nguyen *et al*. (2013)
pKF3-A4		Nguyen *et al*. (2013)
pKF3-ePTEN		Nguyen *et al*. (2014)
pKF3-ePTEN,A4		Nguyen *et al*. (2014)
pKF3-nPTEN		Nguyen *et al*. (2014)
pKF3-nPTEN,A4	nPTEN amplified using primers HuPTEN5BamHI2 and HuPTEN3XhoI2 and inserted into *BglII*/*NheI* pKF3-A4	This study
pKF3-L42R		Nguyen *et al*. (2014)
pKF3-L42R,A4		Nguyen *et al*. (2014)
pKF3-L42R,ePTEN	ePTEN mutated using primers HuPTEN5BamHI2, HuPTEN3XhoI2, ePTEN-L42R-f and ePTEN-L42R-r inserted into *BglII/XhoI* pKF3	This study
pKF3- L42R,ePTEN,A4	ePTEN,A4 mutated using primers HuPTEN5BamHI2, HuPTEN3XhoI2, ePTEN-L42R-f and ePTEN-L42R-r inserted into *BglII/XhoI* pKF3	This study
pKF3-Q17E		Nguyen *et al*. (2014)
pKF3-C124S		Nguyen *et al*. (2013)
pKF3-C124S,A4		Nguyen *et al*. (2013)
pKF3-K13A,A4		Nguyen *et al*. (2014)
pKF3-K13R,A4	A4 mutated using primers HuPTEN5BamHI2, HuPTEN3XhoI2 and PTEN-K13R-f inserted into *BglII/XhoI* pKF3	This study
pKF3-K13R,ePTEN	ePTEN mutated using primers HuPTEN5BamHI2, HuPTEN3XhoI2 and PTEN-K13R-f inserted into *BglII/XhoI* pKF3	This study
pKF3-K289E	hPTEN mutated using primers HuPTEN5BamHI2, HuPTEN3XhoI2, PTEN-K289E-f and PTEN-K289E-r inserted into *BglII/XhoI* pKF3	This study
pKF3-K289E,A4	K289E amplified using primers HuPTEN5BamHI2 and HuPTEN3XhoI2 and inserted into *BglII*/*NheI* pKF3-A4	This study
pKF3-K289E,ePTEN	ePTEN mutated using primers HuPTEN5BamHI2, HuPTEN3XhoI2, PTEN-K289E-f and PTEN-K289E-r inserted into *BglII/XhoI* pKF3	This study
pKF3-ePTEN,ΔPDZ-BM	ePTEN mutated using primers HuPTEN5BamHI2, HuPTEN3XhoI2 and PTEN-ΔPDZ-XhoI-r inserted into *BglII/XhoI* pKF3	This study
pcDNA3.1-PTEN		Nguyen *et al*. (2013)
pcDNA3.1-A4		Nguyen *et al*. (2013)
pcDNA3.1-ePTEN		Nguyen *et al*. (2014)
pcDNA3.1-ePTEN,A4		Nguyen *et al*. (2014)
pcDNA3.1-nPTEN		Nguyen *et al*. (2014)
pcDNA3.1-nPTEN,A4	nPTEN,A4-GFP amplified using primers PTEN-TOPO-f and GFP-TOPO-r inserted into pcDNA3.1	This study
pcDNA3.1-L42R		Nguyen *et al*. (2014)
pcDNA3.1-L42R,A4		Nguyen *et al*. (2014)
pcDNA3.1-L42R,ePTEN	L42R,ePTEN-GFP amplified using primers PTEN-TOPO-f and GFP-TOPO-r inserted into pcDNA3.1	This study
pcDNA3.1-L42R,ePTEN,A4	L42R,ePTEN,A4-GFP amplified using primers PTEN-TOPO-f and GFP-TOPO-r inserted into pcDNA3.1	This study
pcDNA3.1-K13R	PTEN-GFP mutated using primers PTEN-TOPO-f, GFP-TOPO-r and PTEN-K13R-f inserted into pcDNA3.1	This study
pcDNA3.1-K13R,L42R,ePTEN	L42R,ePTEN-GFP mutated using primers PTEN-TOPO-f, GFP-TOPO-r and PTEN-K13R-f inserted into pcDNA3.1	This study
pcDNA3.1-K13R,nPTEN	nPTEN-GFP mutated using primers PTEN-TOPO-f, GFP-TOPO-r and PTEN-K13R-f inserted into pcDNA3.1	This study
pcDNA3.1-K13R,A4	A4-GFP mutated using primers PTEN-TOPO-f, GFP-TOPO-r and	This study

	PTEN-K13R-f inserted into pcDNA3.1	
pcDNA3.1-K13R,L42R,A4	L42R,A4-GFP mutated using PTEN-TOPO-f, GFP-TOPO-r and PTEN-K13R-f inserted into pcDNA3.1	This study
pcDNA3.1-K13R,ePTEN	K13R,ePTEN-GFP amplified using primers PTEN-TOPO-f and GFP-TOPO-r and inserted into pcDNA3.1	This study
pcDNA3.1-K289E	K289E-GFP amplified using primers PTEN-TOPO-f and GFP-TOPO-r and inserted into pcDNA3.1	This study
pcDNA3.1-K289E,A4	K289E,A4-GFP amplified using primers PTEN-TOPO-f and GFP-TOPO-r and inserted into pcDNA3.1	This study
pcDNA3.1-K289E,ePTEN	K289E,ePTEN-GFP amplified using primers PTEN-TOPO-f and GFP-TOPO-r and inserted into pcDNA3.1	This study
pcDNA3.1-K289E,L42R, ePTEN	L42R,ePTEN-GFP mutated using primers PTEN-TOPO-f, GFP-TOPO-r and PTEN-K289E-f and PTEN-K289E-r inserted into pcDNA3.1	This study
pcDNA3.1-K289E,nPTEN	nPTEN-GFP mutated using primers PTEN-TOPO-f, GFP-TOPO-r and PTEN-K289E-f and PTEN-K289E-r inserted into pcDNA3.1	This study
pcDNA3.1-K254R,A4	A4-GFP mutated using primers PTEN-TOPO-f, GFP-TOPO-r, PTEN-K254R-f and PTEN-K254R-r, inserted into pcDNA3.1	This study
pcDNA3.1-K254R	*NheI/NotI* fragment from pcDNA3.1-PTEN inserted into *NheI/NotI* pcDNA3.1-K254R,A4	This study
pcDNA3.1-PTEN (no tag)	PTEN amplified using primers PTEN-TOPO-f and PTEN-NotI-r, inserted into pcDNA3.1	This study
pcDNA3.1-ePTEN (no tag)	ePTEN amplified using primers PTEN-TOPO-f and PTEN-NotI-r, inserted into pcDNA3.1	This study
pcDNA3.1-nPTEN (no tag)	nPTEN amplified using primers PTEN-TOPO-f and PTEN-NotI-r, inserted into pcDNA3.1	This study
pcDNA-ePTEN,ΔPDZ-BM	ePTEN,ΔPDZ-BM-GFP amplified using primers PTEN-TOPO-f and GFP-TOPO-r inserted into pcDNA3.1	This study
pcDNA3.1-hPTEN$_{352\text{-}403}$-YFP-FLAG		Rahdar *et al.* (2009)
pmCherry-PH$_{AKT}$		Gift from T. Inoue*
pLKO.1-shRNA1 NEDD4-1		HiT center, JHU TRCN0000007553
pLKO.1-shRNA2 NEDD4-1		HiT center, JHU TRCN0000007554
pRK5-HA-Ubiquitin		Addgene, 17608
pCI-HA-NEDD4		Addgene, 27002
pCI-neo-mCherry-NEDD4		Addgene, 38316
pCI-neo-FLAG-HAUSP		Addgene, 16655
NES-CFP-FRB	NES-CR was made by inserting an annealed product, NES derived from MAPKK, into CR vector using NheI and AgeI. The sequence of annealing primers are attached (NES(MAPKK)CFP-FRB (ECFP-C1)).	
FRB-CFP-Emerin	FRB-CFP-Emerin was made by replacing GFP of GFP-Emerin (gift from Katherine Wilson) with FRB-CFP (reported in Nat Meth 2005) using NheI and BsrGI.	
NLS-CFP-FRB	NLSx3-CR was made by inserting an annealed product, NLSx3, into CR vector using NheI and AgeI. The sequence of annealing primers are attached (081411).	
YFP-FKBP		Inoue *et al.* (2005) PMID 15908919
YFP-FKBP-βGalΔN		Lin et al. (2013) PMID 23666116

PTEN-YFP-FKBP	PTEN amplified using primers PTEN-N1F and PTEN-N1R and inserted into *EcoRI/BamHI* YFP-FKBP	This study
nPTEN-YFP-FKBP	nPTEN amplified using primers PTEN-N1F and PTEN-N1R and inserted into *EcoRI/BamHI* YFP-FKBP	This study
ePTEN-YFP-FKBP	ePTEN amplified using primers PTEN-N1F and PTEN-N1R and inserted into *EcoRI/BamHI* YFP-FKBP	This study
PTENK13R-YFP-FKBP	PTENK13R amplified using primers PTEN-N1F and PTEN-N1R and inserted into *EcoRI/BamHI* YFP-FKBP	This study
PTENK13R,A4-YFP-FKBP	PTENK13R,A4 amplified using primers PTEN-N1F and PTEN-N1R and inserted into *EcoRI/BamHI* YFP-FKBP	This study
peGFP-C1-PTEN	PTEN amplified using PTEN-XhoI2-f and PTEN-BamHI2-r, inserted into *XhoI/BamHI* peGFP-C1	This study
peGFP-C1-ePTEN	ePTEN amplified using PTEN-XhoI2-f and PTEN-BamHI2-r, inserted into *XhoI/BamHI* peGFP-C1	This study
peGFP-C1-nPTEN	nPTEN amplified using PTEN-XhoI2-f and PTEN-BamHI2-r, inserted into *XhoI/BamHI* peGFP-C1	This study
pmCherry-N1-PTEN	PTEN amplified using PTEN-XhoI-f and PTEN-BamHI-r, inserted into *XhoI/BamHI* pmCherry-N1	This study

Table S2. Primers used in this study

HuPTEN5BamHI2	CTGGGATCCAAATAAAAATGACAGCCATC
HuPTEN3XhoI2	CTCCTCGAGCCGACTTTTGTAATTTG
ePTEN-L42R-f	GATTTCCTGCAGAAGGAAGAGAAGGCGTATACAG
ePTEN-L42R-r	CTGTATACGCCTTCTCTTCCTTCTGCAGGAAATC
PTEN-K13R-f	ATGACAGCCATCATCAAAGAGATCGTTAGCAGAAACAGAA GGAGATA
PTEN-K289E-f	GAGGAAACCTCAGAAGAAGTAGAAAATG
PTEN-K289E-r	CATTTTCTACTTCTTCTGAGGTTTCCTC
PTEN-ΔPDZ-XhoI-r	CTCCTCGAGCCAATTTGTGTATGCTGATCTTC
PTEN-TOPO-f	CACCATGA CAGCCATCAT CAAAG
GFP-TOPO-r	TTACTTGTACAGCTCGTCCATG
PTEN-N1F	AACGAATTCGCCACCATGACAGCC
PTEN-N1R	TAAGGATCCGACTTTTGTAATTTGTGTATGCTG
PTEN-K254R-f	TGTGTGGTGATATCAGAGTAGAGTTCTTCCAC
PTEN-K254R-r	GTGGAAGAACTCTACTCTGATATCACCACACA
PTEN-NotI-r	CTGCGGCCGCTTAGACTTTTGTAATTTGTG
PTEN-XhoI-f	CAACTCGAGATGACAGCCATCATCAAAGAGATCGT
PTEN-BamHI-r	CAAGGATCCCCGACTTTTGTAATTTGTGTATGCTGATCTTC
PTEN-XhoI2-f	CAACTCGAGCTACAGCCATCATCAAAG
PTEN-BamHI2-r	CCAGGATCCTTAGACTTTTGTAATTTGTGTATGC
